# Dimensions of decision difficulty in women’s decision-making about abortion: A mixed methods longitudinal study

**DOI:** 10.1371/journal.pone.0212611

**Published:** 2019-02-22

**Authors:** Jenneke van Ditzhuijzen, Marieke Brauer, Hennie Boeije, Carolus H. C. J. van Nijnatten

**Affiliations:** 1 Department of Social and Behavioral Sciences, Interdisciplinary Social Science, Utrecht University, Utrecht, the Netherlands; 2 Department of Social and Behavioral Sciences, Methods and Statistics, Utrecht University, Utrecht, the Netherlands; University of Toronto, CANADA

## Abstract

Relatively little is known about the ease or difficulty with which women decide to have an abortion, and most research uses single-item measurements. We used a mixed methods approach to combine data from the Dutch Abortion and Mental Health Study (DAMHS, n = 325) with data from a qualitative study about the decision process with a small subsample (n = 69) of the DAMHS study. We used the findings from the qualitative study to develop the Dimensions of Abortion Decision Difficulty [DADD] scale, and tested this scale among a larger sample of women who took part in the second wave of the cohort study (n = 264). Qualitative analyses revealed six dimensions of decision difficulty. The DADD scale was based on these six dimensions. Results from the DADD scale at follow-up reduced these to four dimensions: (1) unrealistic fears about the abortion and fantasies about the pregnancy; (2) decision conflict; (3) negative abortion attitudes; and (4) general indecisiveness. Decision conflict was the only dimension related to previous mental disorders. The findings suggest that the concept of decision difficulty seems multidimensional, rather than unidimensional. On a clinical level, it could be important to separate the more general fears, attitudes, and indecisiveness from strong decision conflict, because the latter might involve pressure of others, lack of decision ownership, and might be related to previous mental health.

## Introduction

In the Netherlands, abortion is free, legal, and available up until 22 weeks of gestation; but at the same time the abortion rate is among the lowest in the world: about 8.6 in 1,000 women aged 15–45 per year have an abortion [1, 2]. Most women who request an induced abortion have already made their decision before they contact the abortion clinic to make an appointment [3, 4]; a US study showed that the median time between pregnancy confirmation and abortion decision was 0 days [5]. However, studies suggested that 25–35% of abortion clients have experienced (some) decision difficulty as to whether to continue or terminate their pregnancy [4, 6, 7]. Verifying whether a woman is certain about her abortion request is a standard procedure in Dutch abortion clinics. As long as she hesitates, the pregnancy cannot be terminated [4]. Even though around 90% of women are satisfied with their decision to terminate an unwanted pregnancy [7], there is a small group of women who feel conflicted and might need options counselling. To identify these women, a deeper understanding of the abortion decision process is required.

Despite its clinical relevance, decision difficulty is infrequently used as a concept in abortion research, and the terminology varies. Other related terms are ‘ambivalence’ [8–10], ‘doubt’ [11], ‘mixed feelings’ [12], ‘confidence’ [13], and ‘opposing feelings’ [9, 10]. The discrepancies in meanings and operationalizations of the concept complicate the interpretation of findings. Furthermore, most studies measured it as a unitary construct (i.e., single-item measures with fixed response options) [11–13]. Decision difficulty can be seen as a healthy response when faced with an unwanted pregnancy, just like immediate decision certainty is a healthy response. Unwanted pregnancy involves a dilemma with two unfavorable options. Some women immediately know what to choose, whereas others need time to weigh their arguments, wishes, and emotions. Decisions may be difficult for some, but once decided, the decision may still be the right one. Earlier research has indeed shown that decision difficulty is not related to decision satisfaction or decision ‘rightness’: most women are satisfied with the abortion, whether they experienced decision difficulty or not [7]. To obtain a better understanding of the abortion decision process and what makes the decision easy or difficult, the first goal of this study was to offer insight in the concept of decision difficulty, using a mixed methods approach.

Some studies have related emotional distress around the abortion to mental disorders [14–15]. In our earlier studies, we found that decision difficulty was unrelated to *post*-abortion development of mental disorders [16], but associated with *pre*-abortion mental disorders [7]. Therefore, a secondary, explorative goal was to explore potential associations of decision difficulty dimensions with a history of mental disorders.

## Materials and methods

### Design

The study is based on both quantitative data of the longitudinal cohort study ‘Dutch Abortion and Mental Health Study’ (DAMHS), and qualitative data of a small subsample of the first measurement of the cohort study. The first quantitative interview was held between April 2010 and January 2011. Of the total sample of women at baseline, we re-approached a subsample of women between November 2010 and June 2011 for a qualitative in-depth interview about the decision process. These qualitative data were used to construct a scale, the Dimensions of Abortion Decision Difficulty [DADD] scale. Between December 2012 and November 2013, which was on average 2.7 years after the baseline quantitative interview, all women who had taken part in the first interview were approached for a follow-up quantitative interview, and the scale was tested among the whole cohort. The inclusion of participants in the three measurements is depicted in S1 Fig.

### Procedure

All interview meetings were held in a private setting, mostly in the respondent’s home. Prior to each interview, the study purposes and procedures were explained, and informed consent was obtained. Confidentiality, anonymous data handling, and the opportunity to withdraw from the interview were ensured. In the in-depth interview, it was stressed that we considered all kinds of reasons, thoughts, and feelings they might have had, as valid and accepted. Participants were encouraged to communicate the salient aspects of their experiences in their own words. All selected interviewers were female and over 25 years old. They received a two-day diagnostic interviewing training, followed up by at least two full test-interviews. The qualitative interviewers had to have extensive experience with qualitative interviewing. Qualitative interviews were audiotaped for transcription, which enabled regular peer supervision and standardization sessions between the interviewers. The mean duration of the interviews was around 2.5 h (baseline), around 1.5h (subsample), and 1.5h (follow-up). For each interview, participants received a gift card of 50 Euros. The study protocols were approved by the Dutch Medical Ethical Committee specialized in mental health METiGG in 2010. In 2012, the METiGG was abolished and the research protocols were transferred to the Central Committee on Research Involving Human Subjects (CCMO).

### Recruitment

The participants were recruited by clinical staff in Dutch abortion clinics. In the Netherlands, the large majority (around 90%) of abortions are performed in these specialized clinics [2]. For representativeness, eight out of sixteen clinics were selected on the basis of geographical location and clinic size, but one clinic could not participate due to management changes and staff turn-over. Shortly after the abortion procedure, staff members asked women to read the research flyer, complete a reply card, and deposit the card in a locked mailbox. The study was restricted to Dutch-speaking women over the age of 18 who terminated an unwanted pregnancy without clear medical indications. To minimize selection bias, staff members were instructed to hand out flyers to every client meeting these criteria, and this was checked using the clinics’ general treatment data. The reply card included a ‘consent-to-contact’ form on one side, and a non-response form on the other side, in case women did not want to be contacted for recruitment purposes. After approximately 2 weeks, women were contacted by telephone or e-mail by the interviewer to check eligibility, capacity to consent (command of the Dutch language, no cognitive impediments), and make an appointment for the interview, in the case that they agreed to participate. Participants were first interviewed 20 to 40 days after the abortion.

### Participants

The quantitative study enrolled 332 Dutch-speaking women 18 to 46 years old, obtaining an abortion (medical or aspiration, until a maximum of 22 weeks) for an unwanted pregnancy, without clear fetal or maternal medical indications. At baseline, seven interviews could not be completed, leaving 325 participants for analysis. At follow-up, 264 participants (81.2%) were re-interviewed. Participant flow and results of a response analysis are described elsewhere [16, 17].

The majority of the women (n = 272) in DAMHS gave consent to be contacted for the qualitative study. Because our main purpose was to investigate the concept of decision difficulty, we approached interested women based on their level of self-reported decision difficulty (high or low). The subsample consisted of 69 women who had had an abortion 4 to 7 months prior to the in-depth interview. The numbers of participants in the low and high decision difficulty groups were 47 and 22, respectively.

### Qualitative interviews (subsample)

The topic list for the qualitative interviews consisted of eight main topics, which are summarized in S1 Appendix. Three of these concerned the circumstances and context of the decision, three considered decision-making, and two were about attitudes and experiences.

### Quantitative measures

#### Decision difficulty

Decision difficulty was measured at baseline with one item (in Dutch: “In hoeverre heeft u over de abortus getwijfeld?”) on a five-point scale from ‘not at all’ to ‘to very large extent’. The Dutch concept of ‘twijfel’ cannot be translated into English directly, it is more neutral and less negative than ‘doubt’, and a bit more serious than ‘think about’. The closest translation in English would be “To what extent did you have difficulty with making the decision to have an abortion?” The respondents were explicitly asked to think about the pre-abortion period. Women were categorized into High Decision Difficulty (HDD) when they scored 4 or 5, and into Low Decision Difficulty (LDD) when they scored 1 or 2.

#### Sociodemographic variables

‘Age’ was measured continuously; women were then categorized into three age groups. ‘Living alone’ was a dichotomous measure of living alone versus living together with a husband or cohabitating with a partner. Similarly, ‘children’ distinguished women with one or more children from those without any children. ‘Education level’ was measured in 8 categories, and then categorized in low = 1 (primary education or lower secondary education) and higher = 0 (higher secondary education and higher professional education). Other measures were: ‘unemployed’ (yes = 1, no = 0); ‘religious’ (yes = 1, no = 0), and ‘non-western ethnicity’: respondents in this category or at least one parent of the respondent were born in Turkey, the Caribbean, Africa, Asia (excluding Japan/ Indonesia) or Latin-America (yes = 1, no = 0), which is the standard definition of Statistics Netherlands (CBS) [18].

#### History of mental disorders

History of mental disorders was assessed at baseline with the Composite International Diagnostic Interview (CIDI) version 3.0, which was developed and validated for the WHO World Mental Health Survey Initiative [19, 20], and adapted to obtain a comparable version in Dutch [21]. The following common mental disorders were included: mood disorders (major depression, dysthymia, bipolar disorder); anxiety disorders (panic disorder, agoraphobia, social phobia, specific phobia, generalized anxiety disorder); and substance use disorders (alcohol/drug abuse and dependence); in addition, the aggregate measure ‘any common mental disorder’ (presence of one or more of all of the measured mental disorders) was included. The prevalence of lifetime mental disorders as measured with the CIDI 3.0 was 43% in the general Dutch population in 2007–9 [21], and 47% in the US population in 2002–3 [20].

#### Dimensions of Abortion Decision Difficulty [DADD] scale

Based on the results of the qualitative analyses (see Results section), six dimensions of decision difficulty were identified. For each dimension, we developed two or three quantitative items, based on actual quotes of participants that were representative of a dimension, adding up to a total of 15 items which could be answered on a scale ranging from ‘Not at all’ to ‘To a very high degree’. This list of items was then discussed with all co-authors and with experts from the field of abortion care. The items were also piloted in the training of the interviewers. The scale was incorporated into the DAMHS interview questionnaire at follow-up. Three items were reverse-scored and recoded before further analyses. Initially, reliability of the whole scale was low (Cronbach’s alpha = .45). Removing three items (‘I was sure I did not want to have any (more) children’; ‘I felt supported by the man involved in the pregnancy’; ‘I did not imagine what the embryo/fetus would look like’) substantially increased the reliability (Cronbach’s alpha = .82). A list of the final 12 questions can be found in S2 Appendix.

### Analysis

First, descriptive statistics were calculated. Chi-square tests were performed to test differences in background and mental health variables between women who experienced high decision difficulty and women who did not. Selection of participants for the qualitative subsample was also based on the single item decision difficulty. Qualitative interviews were transcribed verbatim and were imported in NVIVO9 software for coding and analysis [22]. After the transcripts were reviewed, the material was condensed and meaningful units were extracted. Transcripts were initially coded independently by the second author and a research assistant and compared. Differences in coding were discussed until consensus was reached. Subsequent interviews were coded by the second author and checked by the assistant. Following the constant comparative method [23], new interviews were compared with existing codes to identify similarities and differences. Conceptual saturation was reached when no new categories were generated. By coding and grouping the categories, dimensions of decision difficulty in the interviews with the HDD group emerged. These were systematically compared with the LDD-group. To avoid potential researcher bias, two measures were taken. First, the final codes were discussed within the team of interviewers and supervisors. Second, we discussed our findings in meetings with experts in the field, i.e., representatives of abortion clinics, and welfare workers specialized in counseling women with an unwanted pregnancy. We were able to realize saturation of categories after analyzing 17 interviews in the LDD-group and 15 interviews in the HDD-group. We continued data collection and used subsequent interviews to confirm our observations. In this way, it became clear that the HDD-group differed from the LDD-group on six dimensions, which lead to the development of the DADD scale. Quantitative analyses were performed using SPSS version 24. Reliability and factor analysis using principal component analysis were performed on the DADD scale. In addition, we used correlation and regression analysis to explore which covariates were associated with the dimensions of decision difficulty.

## Results

### Descriptive statistics

Descriptive characteristics of the total baseline sample, as well as the subsample for the qualitative interviews, are displayed in Table 1. The mean age of women in DAMHS was 29.8 years (SD = 7.7). We analyzed whether women experiencing high levels of decision difficulty (HDD) were different from women experiencing low to moderate levels of decision difficulty, in terms of sociodemographics, history of common mental disorders, and abortion related variables. HDD women did not differ from the others in terms of demographic background, but they more often had a history of any mental disorder, *X*_2_ (1, *N* = 264) = 7.68, p = .006, and of substance use disorders, *X*_2_ (1, *N* = 264) = 4.41, p = .04. HDD women also more often had later (second trimester) abortions than the others, *X*_2_ (1, *N* = 264) = 5.62, p = .02.

**Table 1:** Descriptive characteristics (in percentages) of the total baseline sample of DAMHS and a subgroup of women scoring high on preabortion decision difficulty (HDD).

	Total DAMHS sample		Qualitative subsample	
Characteristic	total % (n = 325)	HDD % (n = 81)	total % (n = 68)	HDD % (n = 22)
***Sociodemographics***				
18–24 years	33	32	27	32
25–34 years	36	41	32	41
35–46 years	31	27	41	27
Living alone	55	54	55	49
Children (one or more)	54	48	59	40
Lower education level	21	14	19	23
Unemployed	29	27	29	23
Religious	21	24	13	9
Non-western ethnicity	19	21	12	9
***Previous mental disorders***				
**Any lifetime mental disorder**	68	82**	54	73*
Lifetime mood disorders	41	41	38	50
Lifetime anxiety disorders	40	47	32	50*
Lifetime substance use disorders	23	31*	18	27
***Abortion-related variables***				
Second trimester abortion	6	12*	4	9
Previous abortions	24	25	24	9

* p < .05

** p < .01

Note. Chi-square-tests were performed to compare HDD-groups with the rest of the sample (LDD or moderate DD; the latter category was only present in the Total DAMHS sample).

### Qualitative results

Women in the HDD-group talked about individual as well as contextual dimensions that influenced their abortion decision. Women in the LDD-group did not always address these issues, or mentioned that they did not cause any difficulty deciding. Based on the analyses, we identified six dimensions of decision difficulty.

#### Dimension 1. Positive maternal feelings and wish to have a child

A considerable number of women in the HDD-group felt happy and proud when they discovered that they were pregnant. Many of the HDD-women expressed a wish to have a child, but their unfavorable living circumstances or the status of the partner relationship made them debate whether to continue this pregnancy. They experienced strong ambivalent feelings. One HDD-participant revealed:

It was all so two-sided. This is something I really want, I have a strong desire to have a child, so on the one hand you think ‘I really want it, but on the other hand, it’s his’. And that’s not what I wanted, not this way. I had very contradictory feelings, and of course that made it hard.

Sometimes, women did not mention positive feelings, but described ‘maternal feelings’. The idea of having an abortion felt unnatural to those who described feelings of maternal attachment. Some women thought these positive/maternal feelings were hormonally determined. Some, who already had children and considered their family complete, recognized their maternal feelings from earlier wanted pregnancies. They noted that they could not feel as happy now as during their previous pregnancies. These mothers found the abortion decision hard as they realized that the fetus could grow up like their sons and daughters. They struggled with the question whether it was acceptable to not want this child while earlier pregnancies were welcomed.

Women in the LDD-group less frequently reported positive/maternal feelings in response to the pregnancy compared to women in the HDD-group, even though most LDD-women also had a (future) wish to have a child or already had children and considered their family complete.

#### Dimension 2. Fears around abortion

HDD-women articulated several fears of adverse health or psychological consequences due to abortion. The most mentioned fear was feeling regret. Several women were concerned about potential risks associated with the abortion procedure, e.g., physical damage resulting in decreased fertility. Some (also) feared that they would never get another chance to become a mother. Fears about infertility were mainly prevalent among childless women. One HDD-participant stated:

You can’t help being afraid that they’ll damage something inside you and that you’ll never get the chance to be pregnant again…[…] You think, this could be my only chance.

A few HDD-women were afraid that they would suffer mental health problems due to the abortion. Apart from feared post-abortion consequences, HDD-women often feared adverse experiences (e.g., pain) related to the abortion procedure. In contrast, none of the LDD-women talked about fearing negative consequences due to abortion, although many felt tense prior to the abortion procedure itself. This was, however, not a reason to reconsider their decision.

#### Dimension 3. Negative abortion attitudes

Most HDD-women viewed the pregnancy decision as a choice ‘between life and death’. Many of them understood abortion as taking the life of a human being and therefore found it an objectionable, selfish action. Some were inclined to blame other women for not having taken responsibility for proper contraception. Some believed that other women engage in unprotected sex carelessly and regard abortion as a form of contraception. Consequently, these participants sought to separate themselves from other women having abortions.

Many women set conditions under which they found abortion acceptable. Some found abortion only tolerable for women living in extremely adverse circumstances or in situations in which they could not be held responsible for getting pregnant (e.g., pregnancy resulting from rape, teenage pregnancies). Except for one, none of the participants with a negative abortion view had become pregnant under such circumstances. The discrepancy with their own circumstances was seldom mentioned by participants directly. Rather, these women struggled with feelings of guilt and remorse towards the unborn child and/or towards women with an unfulfilled wish to have a child. They wondered if they were egocentric or were deflecting their responsibility by having an abortion. One HDD-participant stated:

I felt strongly that I was denying life to a child. I kept thinking: ‘You need to take responsibility for your actions. You got pregnant and you’re 38, for goodness sake. You’re not 16 any more. You know how these things work. Dammit, I should have organized some birth control.

Some emphasized that they had no choice other than abortion. They stressed that they would never consider an abortion in other circumstances. It took time to reconcile these women’s feelings of guilt, shame and moral conflict with the abortion decision. As such, a negative abortion view interfered with the decision-making. Similar to HDD-women, women in the LDD-group stressed women’s responsibility for preventing an unwanted pregnancy. Many blamed themselves for not having used proper birth control and sometimes felt ashamed and foolish for getting pregnant. However, these women did not link this responsibility to bringing this pregnancy to full term, whereas women in the HDD-group sometimes argued that a woman should continue her pregnancy if she had failed in contraception use. Some LDD-women spoke of abortion as taking responsibility for preventing a child from being born in unfavorable circumstances. One LDD-participant explained:

If you are in a situation where you really cannot raise a child, I don’t think it’s selfish to do it. You have to make this choice for the child. After all, a child is on the way. And can I offer it anything? Do I want to offer it anything? What is there to offer it? And if there’s nothing to offer and everything is ‘No, No, No’, then it’s not selfish to say, ‘well, I won’t give you life just yet. Because this life that you would get now is not nice’.

#### Dimension 4. Inability to keeping the pregnancy at a distance

Compared to the LDD-group, women in the HDD-group were more often unable to distance themselves emotionally from the pregnancy. This inability to keep the pregnancy at distance manifested itself in several ways. Some of the HDD-women searched more often for information about the development of the fetus, whereas most LDD-women sought only practical information about abortion. A number of HDD-women reported that their attention was drawn to babies, mothers, baby clothes, cuddly toys etcetera. Some of them, especially those experiencing positive feelings, had fantasized about (their future life with) the potential child or about its looks and gender. One HDD-participant stated:

I had images in my head, of a child playing, and all the nice things about it. The feeling that I had always wanted to be a mother—that came up really strongly, like ‘I want to keep it’…And because I wanted to imagine what it would be like to keep it, I kept having thoughts like ‘How would I manage here?’, ‘Where would I put the crib?’, and ‘What about the cats?’ […].

HDD-women also more often considered the fetus as ‘a child’, ‘a baby’, whereas most women in the LDD-group abstracted the fetus and thought of it as ‘it’, ‘a clump of cells’, or ‘something’.

#### Dimension 5. Unsupportive or coercive partner or parents

HDD-women more frequently reported that the sexual partner was unsupportive or had put them under pressure to choose an abortion. A few HDD-women, especially the younger ones, experienced pressure from the parents to have an abortion. None of the women in the LDD-group experienced pressure from others.

In both groups, most partners preferred an abortion. Especially HDD-women in an unstable relationship experienced lack of support from the partner and pressure towards abortion. Initially, these women tried to figure out if they could count on the man as a father and/or partner. The prospect of single motherhood scared them. As the decision process unfolded, their worries about the partner’s role in child-raising increased.

Although less frequently observed, there were a few women in the HDD-group who felt pressured to have an abortion by their partner with whom they already had children. Particularly women who feared abandonment by their partner, expressed that they had no other option. A coercive or unsupportive partner behaved in various ways (e.g., repeatedly emphasizing that abortion is the only solution; stating that he will not care for the child or will leave her if she continues the pregnancy). One HDD-participant stated:

He was shocked that I had such doubts. He hadn’t expected that at all. He was really angry…after I told him, told him in person, I didn’t see him again until the abortion. He was prepared to help me if I got rid of it, then he would come with me…he kept sending negative text messages, always giving reasons for why I couldn’t keep the child and that I would ruin his life and everything…

In the LDD-group, the man’s preference for abortion was seldom experienced as negative. Especially in the case of a stable, lasting relationship, his preference was seen as affirmative of their own choice. The abortion decision was made harmoniously. One LDD-participant stated:

I really felt our family was complete, and luckily my boyfriend felt the same. He had no interest in going back to nappies either. So we agreed on that immediately. The decision was truly a joint one between us.

#### Dimension 6. Indecisiveness as a personality trait

Women in the HDD-group more often characterized themselves as indecisive in general whereas almost none of the women in the LDD-group described themselves as such. Indecisive women found the abortion decision especially difficult given the irreversibility of the choice. They were also afraid to dismiss important matters in considering the options. They wished to be completely sure of their choice, but did not know how and when to reach that point. One HDD-participant stated:

Deciding about a life, that’s something completely different, because your whole life changes. Some decisions can be reversed. But this decision is irreversible. It is Yes or No. There is no middle ground and of course I’m a ‘ditherer’ [indecisive person] anyway.

### Quantitative results DADD scale

To explore whether the six dimensions of decision difficulty would be supported in a larger group, the DADD scale was administered in the full cohort (n = 264). Mean scores on each of the items as well as item-item correlations are displayed in Table 2. We performed principal axis factoring, and used oblique (oblimin) rotation because some of the item-item correlations exceeded .32. The Kaiser-Meyer-Oklin value was .77, which is above the recommended cut-off value of .60; and Bartlett’s test of sphericity was significant, indicating factorability. Inspection of the scree plot revealed a break after four factors. The factor loadings of the items on the four factors are displayed in Table 3. The first factor covered 5 items about the fears and fantasies around abortion versus having a child. The second factor consisted of three items about ‘decision conflict’: women scoring high on this factor had maternal or positive feelings toward the pregnancy, and felt the decision was not completely their own, and had experienced pressure to have the abortion. The third factor consisted of the two items on negative abortion attitudes, and the fourth consisted of the two items on indecisiveness as a personality trait.

**Table 2:** Means (including standard deviations) and item-item correlations for the items of the Dimensions of Abortion Decision Difficulty [DADD] scale.

	M(SD)	Correlations											
		1.	2.	3.	4.	5.	6.	7.	8.	9.	10.	11.	12.
1. Positive/ maternal feelings	2.48 (1.40)	1											
2. Anticipated regrets	2.44 (1.38)	.332**	1										
3. Anticipated infertility	1.62 (1.16)	.051	.320**	1									
4. Afraid of abortion procedure	2.70 (1.39)	.085	.280**	.234**	1								
5. Anticipated MH problems	2.02 (1.20)	.277**	.531**	.230**	.334**	1							
6. Anti-abortion attitude	2.25 (1.52)	.077	.273**	.181**	.169**	.310**	1						
7. Women choose too lightly	2.10 (1.42)	.095	.262**	.168**	.209**	.276**	.610**	1					
8. Fantasizing about child	2.28 (1.36)	.478**	.460**	.151*	.165**	.404**	.146*	.189**	1				
9. My own decision (R)	1.67 (1.16)	.334**	.365**	.139*	.002	.206**	.104	.243**	.271**	1			
10. Felt pressured	1.83 (1.21)	.285**	.402**	.202**	.113	.240**	.141*	.214**	.306**	.646**	1		
11. Difficulty deciding	1.90 (1.28)	.258**	.485**	.265**	.266**	.426**	.253**	.222**	.355**	.257**	.385**	1	
12. Indecisive person	2.19 (1.36)	.185**	.289**	.131*	.253**	.248**	.262**	.194**	.288**	.158**	.212**	.684**	1

* p < .05.

** p < .01.

**Table 3:** Dimensions of Abortion Decision Difficulty [DADD] scale items and factor loadings for the four components.

	Component			
	1	2	3	4
5. Anticipated MH problems	.729			
4. Afraid of abortion procedure	.621			
2. Anticipated regrets	.616			
8. Fantasizing about child	.557			
3. Anticipated infertility	.516			
9. My own decision (R)		-.873		
10. Pressured to have abortion		-.766		
1. Positive/ maternal feelings		-.470		
7. Women choose lightly			.831	
6. Anti-abortion attitude			.801	
12. Indecisive person				-.980
11. Difficulty deciding				-.824

**Note**. Rotation converged in 11 iterations.

### Mental health correlates of decision difficulty

Explorative linear regression analyses were conducted for each of the four factors, with any mental disorder or the three disorder categories as predictors, and age, previous abortions and second trimester abortions as covariates. It was found that the first factor (‘fears and fantasies around abortion and pregnancy’) was only related to second trimester abortions (beta = .18, p = .003). The second factor (‘decision conflict’) was strongly associated with a history of mental disorders (beta = .16, p = .009). This factor was also inversely related to age (beta = -.18, p = .002) and previous abortions (beta = -.18, p = .003): the women scoring high on decision conflict where younger and more often were experiencing their first abortion. The fourth factor (‘general indecisiveness’) was associated with a history of anxiety disorders (beta = .16, p = .01). No further associations were found for the factors with mental health correlates, age, previous abortions or second trimester abortions.

## Discussion

Both the qualitative and quantitative data in this study showed that abortion decision difficulty is a multidimensional concept. The in-depth interviews brought forward six substantive dimensions of decision difficulty. A combination of these individual and contextual dimensions were simultaneously present in decision-making among women who had experienced considerable difficulty deciding. The quantitative testing of these dimensions by means of a newly developed scale, reduced the number of dimensions to four: (1) unrealistic fears and fantasies around pregnancy (termination), (2) decision conflict, (3) negative abortion attitudes, and (4) general indecisiveness.

Although decision difficulty has received relatively little attention in research, the present findings are in line with previous research. Literature suggests that fears and fantasies around abortion are commonly present in the decision-maker’s thinking [24]. Perceived pressure from the partner [6, 25, 26], as well as strong positive feelings towards the pregnancy [9, 10, 27], negative abortion views [13] and experiencing general difficulty in making decisions [13] have been associated with difficulties in decision-making or delay in seeking abortions. These findings underscore the relevance of our newly developed quantitative measure of decision difficulty.

Exploratory analyses showed that women who scored high on the dimension ‘decision conflict’ more often had a history of mental disorders, were younger, and less often had had previous abortions. These women experienced positive maternal feelings, and also more often experienced pressure by others; they expressed that the decision was not entirely their own. Qualitative interviews showed that women experiencing strong decision conflict often do not see themselves as active agents in the decision process, but rather experience that they ‘have no choice’. The quantitative findings show that these women are also more likely to have experienced mental disorders in the past. These results should be interpreted carefully; they do not imply that women with a mental disorder history are less capable of making these decisions. They also do not imply that women with these histories more often have abortions ‘against their will’. They merely show that there is an association between previous mental disorders and decision conflict, which might in turn be related to relationship stability or other important negative life events [16].

The fourth dimension, general indecisiveness, was related to a history of anxiety disorders. Since anxiety disorders often involve ruminating and recurrence of (irrational) thoughts, it seems natural that being indecisive in general is related to this (and not abortion-specific). Unrealistic fears about the abortion and fantasies about the pregnancy, as well as negative abortion attitudes, came about as substantive concepts, but they were unrelated to mental disorders. In other words, these dimensions of decision difficulty are complicating the decision for many women, but they are not indicative of mental disorders.

An interesting finding was that HDD women with negative abortion attitudes would often feel that abortion was only allowed under some strict and severe circumstances, yet almost none of them had become pregnant under these circumstances themselves. They did not mention the discrepancy between their moral beliefs and their own actions, but they did show strong feelings of guilt, shame and remorse, and often felt personally responsible for not having taken contraception properly. A negative abortion attitude, but also unrealistic fears about the abortion, could hamper the decision process. Where unrealistic fears about the abortion procedure can be challenged in counselling, women’s moral beliefs may often be left unaddressed. However, a negative abortion attitude could be indicative of a more difficult decision and abortion process. Fostering openness about abortion (and contraception) in the general population, but also in this specific group, could help women to see things more clearly when faced with an unwanted pregnancy.

Our study aimed to deepen the understanding of the concept of decision difficulty around an abortion. A strength of the study is the rich mixed methods approach, using a longitudinal quantitative dataset as well as in-depth qualitative subset data. Moreover, we used the quantitative data to select cases for the qualitative interview, and subsequently, the qualitative findings served as input for the follow-up quantitative data collection. In this respect, quantitative and qualitative methods not only complemented each other, but they also provided enriching input and further improved the data collection [28].

This study has some limitations as well. First, the retrospective nature of the study may have induced some recall bias. For ethical reasons, it was not possible to interview women before the abortion, therefore, the first measurement of decision difficulty was 3 to 6 weeks post-abortion. Women could have overestimated their own decision difficulty, for reasons of self-preservation, but they could also have underestimated it to remove cognitive dissonance after making the irreversible decision. There is no way to tell whether either type of bias was present, and we found that decision difficulty was fairly constant over time post-abortion. Second, the study sample of the quantitative study was slightly selective in terms of sociodemographics. Compared to the abortion population, the DAMHS quantitative sample was slightly older, higher educated, and more often of Western (Dutch) ethnicity [29]. Thus, the external validity of the scale remains to be investigated. Furthermore, this study was conducted in the Netherlands, where abortion is free and legal. Variables like access to abortion might further complicate the decision process in countries with more restrictive abortion policies, because barriers to have an abortion are also weighed in the decision process.

Our findings have important methodological and clinical implications. At a methodological level, our findings suggest that a unidimensional construct does not do justice to measure the experience of abortion decision difficulty. Single-item measures do not fully capture this complex phenomenon, and important information could be lost. At the clinical level, the findings suggest that women who experience high decision conflict might need more time, attention, and assistance during the decision process. These women are more likely to have had mental disorders in the past, and they more often experience pressure of others to have the abortion. It could be important that counsellors address general fears and ideas about having an abortion, and ease, correct, or normalize these. Decision conflict could also be addressed, to investigate potential coercion or reproductive control [30]. It is important to note that women with strong decision conflict would not necessarily be happier if they chose to carry to term. Decision conflict does not mean that they should not have the abortion, but that they lack decision ownership. Even though it has been found that the way women experience the decision process is not related to future mental disorders when previous mental disorders are taken into account [16], decision conflict could be related to subjective feelings of regret afterwards. To reduce decision conflict, options counselling for these women could specifically focus on agency, so that the women feel they are making their own and the right, or the ‘least painful’, decision considering circumstances, irrespective of the outcome. This ‘choice narrative’ framing could promote a healthy adjustment, because it increases feelings of autonomy [30]. By taking the present findings into account, options counsellors might be better equipped to offer support tailored to women’s specific individual situations.

## Supporting information

S1 Fig Timing and inclusion of participants in the Dutch Abortion and Mental Health Study (DAMHS) and the qualitative subsample. (PDF)

S1 Appendix Topic list for the qualitative interviews (subsample). (PDF)

S2 Appendix Dimensions of Abortion Decision Difficulty (DADD) scale. (PDF)
