# Supplementary material for: Dimensions of decision difficulty in women’s decision-making about abortion: A mixed methods longitudinal study
Source: PLoS One. 2019 Feb 22;14(2):e0212611. doi: 10.1371/journal.pone.0212611 (PMC6386241; doi:10.1371/journal.pone.0212611)

S1 Figure. Timing and inclusion of participants in the Dutch Abortion and Mental Health Study (DAMHS) and the qualitative subsample.

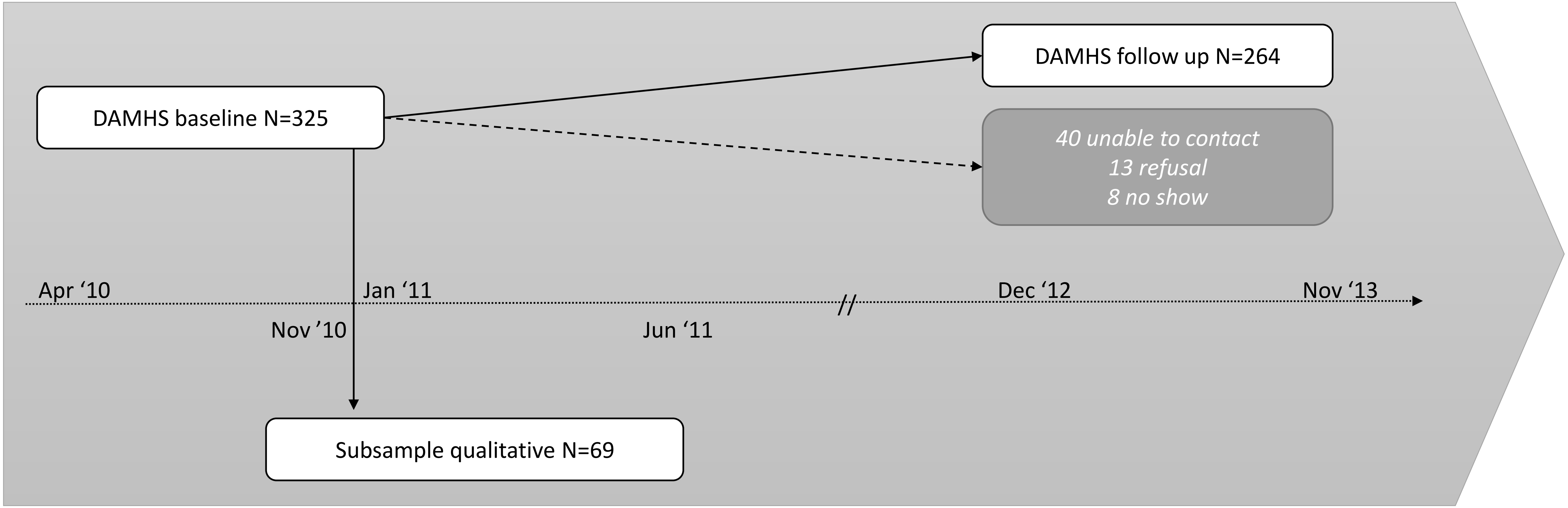

Supplement: S1 Fig — (PDF) [file pone.0212611.s001.pdf]
