# Supplementary material for: Dimensions of decision difficulty in women’s decision-making about abortion: A mixed methods longitudinal study
Source: PLoS One. 2019 Feb 22;14(2):e0212611. doi: 10.1371/journal.pone.0212611 (PMC6386241; doi:10.1371/journal.pone.0212611)
Supplement: S1 Appendix — (PDF) [file pone.0212611.s002.pdf]

**S1 Appendix. Topic list for the qualitative interviews (subsample).**

- Living situation and circumstances of the conception
- Timeline between suspected or discovered pregnancy until the (final) abortion decision
- Motivation for the decision (reasons and feelings pro pregnancy continuation and pro-abortion, decision difficulty)
- Involvement of the sexual partner, family, close relations and professional health care in the decision-making
- Attitudes toward unplanned pregnancy/ abortion
- Experience of the pregnancy
- Level of satisfaction about the decision process
- Decision-making concerning other important life issues
