# Supplementary material for: Dimensions of decision difficulty in women’s decision-making about abortion: A mixed methods longitudinal study
Source: PLoS One. 2019 Feb 22;14(2):e0212611. doi: 10.1371/journal.pone.0212611 (PMC6386241; doi:10.1371/journal.pone.0212611)
Supplement: S2 Appendix — (PDF) [file pone.0212611.s003.pdf]

S2 Appendix. Dimensions of Abortion Decision Difficulty (DADD) scale.

(translated from Dutch)

Please indicate to what extent the following statements about the abortion decision process 2 to 3 years ago are true for you.

1. Even though the pregnancy was unintended, I had positive feelings about being pregnant (like joy, feeling proud, or maternal feelings).
2. I was afraid I would have severe regrets after the abortion.
3. I thought the abortion procedure could induce infertility.
4. I was anxious about having the abortion procedure itself.
5. I was afraid I would develop mental health problems after the abortion.
6. I felt that abortion was in general not justified (save exceptional circumstances).
7. I felt that many women take the choice for abortion too lightly.
8. I fantasized about my life with a child.
9. It was completely my own decision to have an abortion. (*reverse-coded)*
10. I felt pressured (by others) to have the abortion.
11. I have difficulty with decision making in general.
12. I think I am an indecisive person.

Answers on these items are given on the following scale: 1 = not at all; 2 = a little; 3 = somewhat/moderately; 4 = to a high degree; 5 = to a very high degree.
